# A user-friendly method for estimating discrete choice land-use model in a panel data setting

**DOI:** 10.1016/j.mex.2024.102841

**Published:** 2024-07-05

**Authors:** Man Li, Asif Ahmed Khan

**Affiliations:** Department of Applied Economics, Utah State University, Logan, UT 84322, USA

**Keywords:** Nonlinear panel data analysis, Stratified random sampling, Logit model, Autocorrelation, Land-use dynamics, Stratified sampling dynamic land-use model.

## Abstract

Land-use modeling stands as a pivotal tool in shaping sustainable development policies. With the rapid advancement of remote-sensing technology and the widespread adoption of satellite imagery-based land cover products, these datasets have emerged as primary sources for understanding land-use dynamics due to their high spatial and temporal resolutions. Yet, it remains challenging to effectively integrate such rich panel data into nonlinear econometric land-use models. This paper introduces a method to seamlessly incorporate land cover panel data into econometric models, enabling comprehensive utilization of temporal information within a single framework.

- By capturing dynamic land-use patterns, the method enhances prediction accuracy while mitigating issues such as autocorrelated error terms commonly encountered in panel data analysis.
- The method is straightforward to implement and applicable to many nonlinear models, making it particularly suitable for datasets with large sample sizes.

## Graphical abstract

**Figure:**
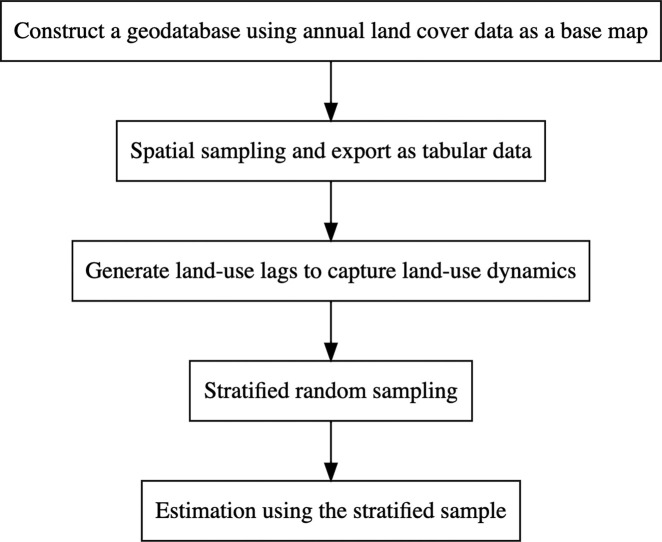

Specifications table

**Table:** 

Subject area:	Economics, Econometrics and Finance
More specific subject area:	Econometric land-use model
Name of your method:	Stratified sampling dynamic land-use model
Name and reference of original method:	N/A
Resource availability:	N/A

## Background

Land-use modeling is essential for developing policies that promote land-based sustainable development and has been widely used in many areas such as carbon sequestration, greenhouse gas emissions, ecosystem enhancement, agricultural land protection, and water resources management. Traditionally, land-use models have mainly relied on aggregate-level area data at the administrative unit level (e.g., [1,2]) or the U.S. National Resources Inventory (e.g., [3,4]). With the rapid development of remote-sensing technology and the widespread use of satellite imagery land cover products, satellite imagery-based land cover products have become the main data source for modeling land use and land-use change (LULUC) in the past two decades. One advantage of these land cover products is their high spatial and temporal resolutions. However, how to effectively incorporate these rich panel data into econometric land-use models remains challenging.

This is because the main advantage of panel data over cross-sectional data in regression is the inclusion of individual fixed effects to absorb time-invariant factors, whether observed or unobserved. In a linear regression model, individual fixed effects are easily eliminated by subtracting the individual mean from each observation (or by first difference). Yet econometric land-use models are theoretically developed based on random utility theory and empirically estimated using discrete-choice models. Since discrete-choice models are nonlinear models commonly estimated using maximum likelihood (ML) methods, it is difficult to exploit land cover panel data by simply including time-invariant individual fixed effects as explanatory variables in the regression. This challenge can be illustrated using both logit and probit models, where fixed-effects estimators are inappropriate.^1^

In addition to fixed effects estimators, panel data can be estimated in a (multinomial) logit model in the same way as pooled cross-sectional data if the unobserved factors affecting decision makers are independent over time [6]. But this is a strong assumption which is likely to be violated in reality. Despite the abundance of remote-sensing data available to measure land use, spatially disaggregated socioeconomic variables that can be used to explain decision-making processes remain limited. This increases the chance of violating assumption. Unlike logit models, probit models allow autocorrelated errors in panel data; but the models are only suitable for strictly discrete choices but not for continuous outcomes of land use measured as proportions. Furthermore, there are no closed-form expressions for high-dimensional integrals (e.g., when the number of choices is greater than three) in multinomial probit models. It is also recommended to use random effects (mixed) logit or probit models to estimate panel data as such models allow time-dependent unobserved factors. But random effects logit/probit models are unattractive too because no simple estimator is available [5] (p. 490).

In practice, when multi-period data are available, many econometric land-use models often estimate land-use changes separately for specific time intervals (e.g., five years) and then use the latest time interval to predict future land-use change (e.g., [3,[7], [8], [9], [10], [11]]). However, the estimation results may be sensitive to the choice of time interval, especially when the land cover or land use data for the selected years are anomalies.^2^ Furthermore, if the aim is to predict future land-use changes, estimating multiple land-use change periods individually would add significant effort but would not contribute much to drawing conclusions.

### Method details

In this paper, we develop a method that employs a two-step sampling strategy (spatial sampling and stratified sampling) while explicitly modeling land-use dynamics to effectively incorporate land cover panel data into econometric land-use models. This method has five advantages. First, it allows the use of information from all time period in a single model, thus avoiding the selection of time intervals. This distinguishes the method from many earlier studies that estimate land-use changes for specific time intervals separately [3,[7], [8], [9], [10], [11]]. Second, the method allows capturing land-use dynamics and some unobserved time-invariant location-specific factors by including land-use lags, thereby significantly improving model prediction accuracy. Although some early studies informally capture the dynamics by separating models by different initial (discrete) land uses [3,4,10], this strategy is not applicable when land use is measured as continuous shares, which is common in observations at the parcel or administrative unit level. Several other studies use lagged crop shares to model crop choices [11,12,16], but these models are cross-sectional in nature. The third advantage of our method lies in its stratified sampling research design, which mitigates the issue of autocorrelated error terms in panel data analysis, thus lowering the risk of biased estimates. The fourth advantage of this approach is its two-step sampling strategy, which significantly reduces the sample size—a crucial factor for estimating nonlinear regression models with very large datasets. Fifth, this method is easy to implement as it addresses the afforementioned econometric issues common in spatial panel data settings at the research design stage rather than during model estimation. This makes the approach much more user-friendly compared to earlier land-use models in the literature.

It is important to note that although some aforementioned previous studies have partially addressed certain econometric issues, there is a lack of systematic discussion of these strategies with broader applicability. While we demonstrate here its application in a multinomial logit land-use model setting, the method is readily applicable to other nonlinear models of panel data involving series autocorrelation in unobserved factors. It is especially suitable for data with large sample sizes, which make the estimation of nonlinear models computationally intensive.

### Modeling setup

Economic theory suggests that in each time period, landowners choose the use of land that yields the highest (expected) net economic return among a number of mutually exclusive alternatives [14,15]. Net economic return refers to the one-period economic return of land use minus annualized one-time land-use conversion costs from the initial use to the selected use [16]. But in reality, land-use conversion costs do not exist, and researchers often use initial land use as inertia to capture conversion costs [3,4,8,12,13,17]. Including initial land use can also help absorb many unobserved location-specific characteristics associated with land use and significantly improve the accuracy of model predictions.

This decision-making process can be formulated within a framework of random utility theory and estimated using a discrete-choice model. At time *t*, for land parcel *n*, land use *j* is selected if and only if $R_{\mathrm{njt}}>R_{\mathrm{nkt}},\;j,k=1,\cdots ,J\;\text{and}\;j\neq k$*.* The term $R_{njt}$ represents the net economic return of land use *j* for land parcel *n*, at time *t; k* represents any alternative use other than *j*. Empirically, $R_{njt}$ can be specified as

(1) $$R_{njt}=y_{nt-1}\alpha_{j}+X_{nt}\beta_{j}+\tau_{t},\;\forall \;n,j,t,$$

where $y_{nt-1}$ represents a land-use vector with (*J*−1) dimension of land parcel *n* at time *t*−1, representing the initial land use during the land-use change period from *t*−1 to *t*. If the land-use data are discrete choices, then $y_{nt-1}$ is a set of categorical variables; if the land-use data are aggregate areas, then $y_{nt-1}$ is a set of continuous variables representing the share of each use on parcel *n*. Note that $y_{nt-1}$ has a dimension of *J*−1 rather than *J* because one choice is eliminated to avoid perfect collinearity. The term $X_{nt}$ is a set of variables thought to influence land-use change; these variables may or may not change over time. But for notational convenience, the uniform $X_{nt}$ is used here. Some variables in $X_{nt}$ may also be alternative specific, depending on the research context and available data. However, these details do not affect the modeling setup and the methodology developed in this paper. The term $\tau_{t}$ represents time fixed effects and is used to capture unobserved time-varying factors common to all parcels. The inclusion of *τ*_*t*_ is optional and depends on the study and data context.

This type of discrete-choice model can be estimated in a logit-type regression model. Despite the relatively strict assumption of independence of irrelevant alternatives, logit-type regression has become the main method for estimating land-use models because it has a closed-form choice probability formula and is easy to implement and interpret [6].^3^ Specifically, the probability of selecting land use *j* on parcel *n* at time *t* takes the form of a logistic function,

(2) $$P\left(y_{nt}=j\right)=\frac{\text{exp}\left(R_{njt}\right)}{\sum_{k=1}^{J}\text{exp}\left(R_{nkt}\right)},\;\forall \;n,j,t,$$

wher*e* $y_{nt}$ represents the land-use choice on parcel *n* at time *t* and $R_{njt}$ is defined in Eq. (1).

### Research design

Suppose the objective is to explore factors in affecting land-use change over *T* years (*T* > 2) in a study area for which annual land cover GIS data are available in raster. The workflow is summarized in the graphical abstract and data sampling proceeds as follows.

First, construct a geodatabase including annual land cover data and other GIS data (i.e., $X_{nt}$) thought to influence land-use change. These GIS data are likely to be geophysical variables such as temperature, precipitation, elevation, land quality (or soil type), etc. or socioeconomic variables such as road density. The land cover data serve as the base maps. Other GIS data are added to the base maps by changing their spatial resolutions to be the same as the base maps.

Second, spatial sampling is performed by selecting a centroid grid on a square, which can be a 5 × 5 grid, a 9 × 9 grid, or even larger. This step, also called as spatial scheme, developed by Besag [18], ensures that the selected grids are far enough apart to reduce potential spatial autocorrelation [19,20]. After spatial sampling, the same *N* grids are selected for each period, resulting in a total of *N* × *T* grids. Then, the spatially sampled geodatabase is processed into a comprehensive point shapefile with *N* observations for each period. Then, the shapefile is exported to tabular data with a total of *N* × *T* observations.

Third, generate the one-period land-use lag for time *t* = 2*,* …, *T* and add this variable to the tabular data. This can be done in the *R* programming language using dplyr::group_by() to sort each grid in ascending order by time and then using stats::lag() to create the lags. Note that for the first time period with *t* = 1, the land-use lag is not available. Therefore, the land cover data for the first time period can only be used as lagged land use (i.e., initial land use) for the second time period. Adding land-use lag reduces the sample size to *N*×(*T*−1) observations, where *t* = 2, …, *T.*

Fourth, stratified random sampling is further performed on the selected *N*×(*T*−1) panel dataset, where each selected grid *n* is treated as a stratum (*n* = 1, …, *N*). Therefore, in each stratum, there are *T*−1 time points and simple random sampling is performed by selecting one of *T*−1 time points for each stratum.^4^ After stratified random sampling, the sample size is further reduced to *N*. This step ensures that each grid (i.e., each location) is selected only once and that no grids are observed at multiple time points. This stratified sampling strategy allows the creation of a data sample that remains representative for the *T*−1 land-use transition periods. More important, it excludes the possibility that the same land-use observations appear as both outcome and explanatory variables, which would lead to endogeneity problems and biased point estimates.

Finally, the stratified random sample with *N* observations may be estimated using discrete-choice models, such as logit-type regression (logit regression, multinomial logit regression, nested logit regression, or conditional logit) or probit-type regression.

## Method validation

To validate the method developed in the previous section, we estimate a multinomial logit land-use model via the function multinom() from the nnet package in the *R* programming language [21] to understand the factors influencing land-use change on private agricultural land in Weber County, Utah, USA, from 2017 to 2022. Further details on the code and data used in the validation are provided in the online supplementary materials. Annual land cover data are collected from the Cropland Data layers at 30-meter resolution [22]. Land-use choice is classified into five categories: alfalfa, non-alfalfa hay, grain crops, pasture, and fallow. Table 1 presents the comparison of the actual land-use transition matrices from the previous period *t*−1 to the current period *t* (*t* = 2018, …, 2022), with and without stratified random sampling. It shows that the stratified sample remains representative of the original non-stratified sample.

**Table 1:** Land-use Conversion Matrices from *t*-1 to *t* (*t* = 2018, 2019, 2020, 2021, 2022).

	Land use in *t*					
Land use in *t***−**1	Alfalfa	Hay	Grain Crops	Pasture	Fallow	Total
*Panel A: Stratified sampling (N = 16,438)*						
Alfalfa	0.407	0.021	0.018	0.006	0.002	0.453
Hay	0.016	0.155	0.005	0.014	0.001	0.190
Grain Crops	0.023	0.005	0.086	0.000	0.003	0.116
Pasture	0.009	0.018	0.002	0.201	0.001	0.230
Fallow	0.001	0.001	0.003	0.001	0.005	0.010
Total	0.455	0.199	0.113	0.222	0.012	1.000
*Panel B: Non-stratified sampling (N = 82,190)*						
Alfalfa	0.406	0.021	0.017	0.005	0.001	0.451
Hay	0.014	0.158	0.004	0.013	0.001	0.190
Grain Crops	0.023	0.005	0.087	0.000	0.003	0.117
Pasture	0.010	0.017	0.002	0.201	0.001	0.230
Fallow	0.001	0.001	0.003	0.001	0.005	0.011
Total	0.454	0.202	0.112	0.221	0.011	1.000

Note: The number in each cell is the number of observations of a specific land-use conversion divided by the sample size and represents the proportion of that specific land-use conversion in the sample. In each panel, the sum of all cells is 1.

The model is specified as in Eqs. (1) and (2). The term $X_{nt}$ includes MODIS-Enhanced Vegetation Index (EVI), annual mean temperature and its squared, growing season (April to September) precipitation and its squared, and elevation. Table 2 shows the comparison of model performance with and without stratified random sampling in terms of prediction accuracy and time required to fit the model, where columns 1 and 2 correspond to the stratified random sampling proposed in this paper, and columns 3 and 4 correspond to the results from a much larger sample without stratified random sampling. In each sampling strategy, the first column corresponds to the model specification that includes one-period land-use lags (i.e., columns 1 and 3), and the second column corresponds to the model specification that does not include land-use lags (i.e., columns 2 and 4).

**Table 2:** Comparison of Model Performance.

	Stratified random sampling		Non-stratified sampling	
	Including land-use lag (1)	Excluding land-use lag (2)	Including land-use lag (3)	Excluding land-use lag (4)
No. of observations	16,438	16,438	82,190	82,190
McFadden's *R*^2^	0.672	0.285	0.678	0.283
Overall accuracy	0.871	0.631	0.873	0.629
User CPU time (s)	1.957	1.221	8.869	6.266
System CPU time (s)	0.011	0.011	0.050	0.020
Elapsed time (s)	1.873	1.231	8.919	6.285

Findings from Table 2 can be summarized into two points. First, including land-use lags as inertia significantly improves model performance in both McFadden's pseudo *R*^2^ and the overall prediction accuracy. Specifically, regardless of whether the data is stratified sampled or not, pseudo *R*^2^ increases from approximately 0.28 to 0.67–0.68, and the overall accuracy of model predictions increases from approximately 0.63 to 0.87. Second, estimating with the stratified sample significantly reduces the time required to fit the model. For example, running the dynamic model with land-use lags using the stratified sample takes 1.873 s (actual elapsed time), much faster than running the same model but using the non-stratified sample (8.919 s); the latter takes almost five times as long as the former. Importantly, this reduction in running time (due to reduced sample size) does not compromise the model performance. Model performance of both (stratified and non-stratified) samples is almost identical regardless of whether land-use lags are included or not. More details about the prediction accuracy of the preferred model (stratified sampling model with land-use lags) are provided in Appendix Tables A1 and A2.

In addition, we compare the estimated marginal effects of explanatory variables on the probabilities of land-use choices between the stratified sampling model (Panel A, Appendix Table A3) and the non-stratified sampling model (Panel B, Appendix Table A3).^5^ The marginal effects are evaluated at the mean values in each sample as generally recommended in the literature. The signs and magnitudes of the marginal effects between the two models are also very close. The above findings shows that the stratified sample retains the representativeness of the original sample and can produce robust estimation results.

## Limitations

Not applicable.

## Ethics statements

Our work did not involve human subjects, animal experiments, or data collected from social media platforms.

## CRediT authorship contribution statement

**Man Li:** Conceptualization, Methodology, Validation, Supervision, Writing – original draft, Writing – review & editing. **Asif Ahmed Khan:** Methodology, Software, Validation, Formal analysis, Investigation.

## Declaration of interests

The authors declare that they have no known competing financial interests or personal relationships that could have appeared to influence the work reported in this paper.

## Data Availability

I have shared my research data and code in the attached files.

## Appendix Supplementary materials

Image, application 1

Image, application 2

Image, application 3

## Footnotes

1 Estimation of a fixed-effects logit model requires the estimation of condition ML function which involves complex formulas. Furthermore, fixed effects in a logit model cannot be estimated directly, and therefore the marginal (or partial) effects on response probabilities cannot be estimated [5] (pp.491–492). As for fixed-effects probit models, the estimation is computationally difficult; moreover, it introduces incidental parameter problems and biased point estimates (see [5], pp. 483–484 for more discussion).

2 This may occur in the presence of socioeconomic shocks (e.g., price shocks may affect crop selection) or inaccuracies in converting satellite images into land cover data (e.g., clouds may affect the quality of satellite images).

3 Although machine learning methods have been increasingly used in land-use modeling as an alternative to logit-type regression, the methods have their own limitations. First, machine learning methods are data-driven and lack the theoretical foundation to explain the economic mechanisms behind LULUC. Second, machine learning methods are not suitable for understanding the marginal effects of changes in any given factor on land-use change. Third, machine learning methods are more likely to suffer from potential overfitting problems.

4 In logit-type regression, it is difficult to interpret coefficient estimates due to the nonlinear nature of the model. It is common practice is to report the marginal (or partial) effects of the explanatory variables on the probability of each choice (including the reference choice).

5 If the panel data are unbalanced (e.g., some pixels have missing time points), we recommend retaining the missing time points when performing stratified sampling to ensure that all observations are randomly selected with equal probability.
