# Corrigendum to “A user-friendly method for estimating discrete choice land-use model in a panel data setting” [MethodsX 13 (2024) 102841]

**DOI:** 10.1016/j.mex.2024.102869

**Published:** 2024-07-19

**Authors:** Man Li, Asif Ahmed Khan

**Affiliations:** Department of Applied Economics, Utah State University, Logan, UT 84322, USA

The authors regret to exchange to contents of footnotes 4 and 5. The correct footnote 4 is “If the panel data are unbalanced (e.g., some pixels have missing time points), we recommend retaining the missing points when performing stratified sampling to ensure that all observations are randomly selected with equal probability.” The correct footnote 5 is “In logit-type regression, it is difficult to interpret coefficient estimates due to the nonlinear nature of the model. It is common practice to report the marginal (or partial) effects of the explanatory variables on the probability of each choice (including the reference choice).”

The authors would like to apologize for any inconvenience caused.

